# Deletion of the Complement C5a Receptor Alleviates the Severity of Acute Pneumococcal Otitis Media following Influenza A Virus Infection in Mice

**DOI:** 10.1371/journal.pone.0095160

**Published:** 2014-04-16

**Authors:** Hua Hua Tong, Garrett Lambert, Yong Xing Li, Joshua M. Thurman, Gregory L. Stahl, Kelsey Douthitt, Caitlin Clancy, Yujuan He, Andrew S. Bowman

**Affiliations:** 1 Department of Otolaryngology, College of Medicine and Public Health, The Ohio State University, Columbus, Ohio, United States of America; 2 Department of Medicine, University of Colorado Denver, Aurora, Colorado, United States of America; 3 Center for Experimental Therapeutics and Reperfusion Injury, Department of Anesthesiology, Perioperative and Pain Medicine, Harvard Medical School, Boston, Massachusetts, United States of America; 4 Department of Clinical Laboratory Medicine, Chongqing Medical University, Chongqing, Sichuan, P. R. China; 5 Department of Veterinary Preventive Medicine, College of Veterinary Medicine, The Ohio State University, Columbus, Ohio, United States of America; Albany Medical College, United States of America

## Abstract

There is considerable evidence that influenza A virus (IAV) promotes adherence, colonization, and superinfection by *S. pneumoniae* (Spn) and contributes to the pathogenesis of otitis media (OM). The complement system is a critical innate immune defense against both pathogens. To assess the role of the complement system in the host defense and the pathogenesis of acute pneumococcal OM following IAV infection, we employed a well-established transtympanically-induced mouse model of acute pneumococcal OM. We found that antecedent IAV infection enhanced the severity of acute pneumococcal OM. Mice deficient in complement C1qa (*C1qa^−/−^*) or factor B (*Bf ^−/−^*) exhibited delayed viral and bacterial clearance from the middle ear and developed significant mucosal damage in the eustachian tube and middle ear. This indicates that both the classical and alternative complement pathways are critical for the oto-immune defense against acute pneumococcal OM following influenza infection. We also found that Spn increased complement activation following IAV infection. This was characterized by sustained increased levels of anaphylatoxins C3a and C5a in serum and middle ear lavage samples. In contrast, mice deficient in the complement C5a receptor (C5aR) demonstrated enhanced bacterial clearance and reduced severity of OM. Our data support the concept that C5a-C5aR interactions play a significant role in the pathogenesis of acute pneumococcal OM following IAV infection. It is possible that targeting the C5a-C5aR axis might prove useful in attenuating acute pneumococcal OM in patients with influenza infection.

## Introduction

Otitis media (OM) is one of the most common diseases among infants and young children worldwide. Children with OM are at an increased risk of secondary bacterial infection following an upper respiratory viral infection [1], [2], [3]. Epidemiological, clinical, and experimental evidence have long supported an association between antecedent influenza A viral (IAV) respiratory tract infection and OM. IAV promotes *Streptococcus pneumoniae* (Spn)-induced OM [4], [5], [6], [7]. Multiple molecular mechanisms have been implicated in IAV–Spn synergism [8], [9]. We and others have reported that IAV enhances Spn nasopharyngeal colonization, which is a prerequisite for the development of OM [6], [7]. Viral compromise of eustachian tube (ET) mucosal integrity results in impaired bacterial clearance and negative pressure in the middle ear in the chinchilla OM model [10], [11], [12]. Virus-modulated host immune and inflammatory responses also play a significant role in pneumococcal OM [13], [14]. Two recent reports suggest that mice pre-colonized with Spn are more susceptible to IAV [14], [15]. They found that the inflammatory response to IAV infection mediates pneumococcal replication in the middle ear cavity in an infant mouse model of OM [14]. Much more needs to be learned about the interactions between IAV and Spn and their combined role in the host immune response, the host defense, and the pathogenesis of OM.

The complement system is a major component of the host innate immune defense system against infection [16]. Activation of the complement system in response to invading pathogens is mediated through the classical, alternative, and lectin pathways. Activation results in C3 cleavage, release of anaphylatoxins C3a and C5a, and the formation of a membrane attack complex (C5b-9) that lyses target cells. Cleavage of C3 generates the key opsonins, C3b and iC3b. These proteins tag the pathogens for phagocytosis [16]. We previously reported that the complement system is activated in the middle ear during acute pneumococcal OM [17], [18]. Both the classical and alternative pathway activations were shown to play important roles in complement mediated opsonophagocytosis of Spn in the middle ear of mice. Other investigators have demonstrated that C3 is required for protection from influenza infection, adequate viral clearance, and inflammatory cellular infiltration [19].

The complement system has been described as a double-edged sword because of its dual role in host defense and pathogenesis of disease [16]. Generation of anaphylatoxins C3a, C4a, and C5a that is not specific for invading pathogens can contribute to the pathogenesis of various acute and chronic inflammatory diseases [20]. A recent report shows that complement C5 activation during Influenza A infection contributes to neutrophil recruitment and lung Injury in mice [21]. Inhibition of C5 activation reduces excessive inflammatory reactions associated with the severe forms of IAV infections [21]. C5a also plays a significant role in both inflammatory and immune effects through interactions with C5a receptors, C5aR (CD88) and a second receptor, the C5a-like receptor 2(C5L2). Expression of C5aR has been reported on many cell types including several myeloid cells (*e.g.*, neutrophils, monocytes/macrophages, and mast cells) and non-myeloid cells such as epithelial and endothelial cells [22]. Interaction of C5a/C5aR leads to the induction of local inflammation through cellular degranulation, increased vascular permeability, and leukocyte recruitment to the site of injury or infection [22]. However, much less is known about the role and extent of complement activation during acute pneumococcal OM following IAV infection.

In the current study, we utilized a well-established transtympanically-induced acute pneumococcal OM mouse model following an IAV infection to investigate how complement activation in this setting affects the middle ear defense against acute pneumococcal OM. The studies were conducted in four groups of mice that differed in their ability to activate various components of complement. Mice deficient in C1qa (*C1qa ^−/−^*) are unable to activate complement through the classical pathway. Mice deficient in factor B (*Bf ^−/−^*) are unable to activate complement through the alternative pathway. Mice deficient in C5aR are unable to interact with C5a. Parental wild-type (WT) mice served as controls. The mice were inoculated intranasally with IAV and then challenged transtympanically 7 days later with Spn type 6A. All cohorts were observed over a 14-day period after inoculation with Spn. These studies revealed that both the classical and alternative pathways are critical components of the otological innate immune defense against Spn following IAV infection. We were also able to show that complement activation promoted increased production of C5a. This was associated with more severe inflammation in Spn-induced OM following IAV infection. In contrast, deficiency in C5aR attenuated the generation of inflammatory mediators and accelerated Spn clearance in this model.

## Materials and Methods

### Infectious agents

Spn type 6A (EF3114) with a predominant transparent phenotype was provided by B. Anderson, Department of Clinical Immunology, University of Göteborg, Sweden [23]. The growth conditions and inocula were prepared as previously described [17]. Influenza virus strain A/Puerto Rico/8/34 H1N1 (A/PR/8/34) was obtained from Charles River Laboratories Avian Vaccine Services (North Franklin, CT). The HA titer of the purchased virus was 1∶65,536 per 0.05 ml, and the 50% embryo infectious dose (EID50) titer was 10^9.5^/ml.

### Mice

Eight to 12-week-old male or female C57BL/6 mice were used in this study. C57BL/6 mice homozygous for gene deficiencies of C1qa (*C1qa^−/−^*) and factor B (*Bf ^−/−^*) were generated as previously described [24], [25]. *C1qa ^−/−^* and *Bf ^−/−^* mice were backcrossed at least 9 generations onto the C57BL/6 background. Age and sex matched C57BL/6 (WT) mice, used as controls, were obtained from the Jackson Laboratories (Bar Harbor, ME). *C5ar1^−/−^* mice with genetic background of BALB/c, and BALB/c (WT) mice were obtained from The Jackson Laboratory. Blood samples from 3–5 mice with the same genetic background were obtained by cardiac puncture. Single use aliquots of the sera were stored at −70°C. All study procedures were approved by The Institutional Animal Care and Use Committee at the Ohio State University.

### Mouse models of acute pneumococcal OM without prior IAV Infection

Acute pneumococcal OM was induced by direct bilateral transtympanical inoculation of the middle ear, as previously described [26]. Briefly, mice were anesthetized by intraperitoneal injection with ketamine hydrochloride (20 mg/kg of body weight) and xylazine (5 mg/kg). Acute pneumococcal OM was then produced by the inoculation of 5 µl of a suspension containing approximately 500 CFU of Spn type 6A in sterile pyrogen-free saline. A control cohort of five mice was sham inoculated with 5 µl of diluent alone. On days 1, 3, 7, and 14 post Spn infection, mice were anesthetized and then sacrificed. The middle ear spaces were lavaged to quantitatively determine titers of Spn as previously described [17]. Blood for serum samples was collected via cardiac puncture and quantitatively cultured to determine bacterial dissemination. The middle ear lavage and blood samples were cultured overnight at 37°C on Columbia CNA agar plates in an incubator supplemented with humidity and 5% CO_2_. The number of CFU per milliliter was determined by a standard dilution assay and plate counting.

### Antecedent Influenza A virus infection

Mice were infected intranasally (i.n.) with a sublethal dose of 10^2.5^ EID_50_ of A/PR8/34 influenza virus in 25 µl 7 days prior to transtympanical inoculation with about 500 CFU of Spn type 6A. This influenza dose was optimized to give ∼10% weight loss in WT mice during the peak of infection and allowed for recovery without incidence of mortality. The viral titers (PFU/ml) in the tracheonasal and middle ear lavage samples were determined by plaque assay on Madin-Darby Canine Kidney (MDCK) cell monolayers as previously published [27].

### Histology

Six temporal bones from each cohort were removed immediately after sacrifice at 24, 48 and 72 h post Spn challenge. The samples were processed for hematoxylin and eosin staining as described previously [17].

### Immunohistochemistry

Temporal bones from 3 mice in each cohort were removed immediately after sacrifice at 24 h post-challenge. The samples were processed as described previously with minor modifications [18]. The middle ear sections were deparaffinized and rehydrated through histoclear and graded alcohol series. The endogenous peroxidase activity was blocked with 0.3% H_2_O_2_ in 0.1 M phosphate-buffered saline (PBS) (pH 7.4) and the sections were incubated with 0.05% trypsin solution (Invitrogen) at 37°C for 20 min to unmask antigens. The sections were then blocked with PBS (pH 7.2) containing 1% bovine serum albumin (BSA), 5% donkey serum, and 0.3% Triton X-100 for 1 h. The sections were then incubated at 4°C overnight with primary antibodies: goat-anti mouse C5aR polyclonal antibody (1∶200, Santa Cruz Biotechnology, San Diego, CA), goat-anti mouse C3 (1∶200, MP Biomedicals, Solon, OH), or rabbit anti mouse C5b-9 (1∶500, abcam). Samples were washed and incubated with 1∶500 dilutions of secondary antibodies conjugated to DyLight 488 or DyLight 594 (Jackson ImmunoResearch Laboratories, West Grove, PA) at room temperature for 1 h followed by incubation with DAPI (1∶10,000, Invitrogen) at room temperature for 2 minutes. The sections were mounted with Vectashield Mounting Medium (VectorLaboratories, Burlingame, CA). Immunostained samples were examined with an Olympus Flowview 1000 laser scanning confocal microscope.

### Quantitation of complement and cytokine proteins in the middle ear lavage and serum samples by ELISA

The middle ear lavage fluid samples from mice were centrifuged at 500×g and the supernatants were aliquoted and frozen at −70°C. Concentrations of C3, C3a, C5a, CXCL1(KC), interleukin 6 (IL-6), and monocyte chemoattractant protein-1 (MCP-1) were determined using commercial ELISA kits (GenWay Biotech, San Diego, CA; Quantikine; R&D Systems, Minneapolis, MN), according to the manufacturer's instructions.

### Statistical analysis

Data are presented as the mean ± the standard error of the mean (S.E.M.) or standard deviation of the mean (S.D.) as indicated. Data were analyzed using SigmaStat (SPSS Inc., Chicago, IL). One-way ANOVA and the Holm-Sidak or Dunn's methods were used for the statistical analysis and pair-wise multiple comparisons. Mann-Whitney U test was used to compare the differences in Spn concentrations in middle-ear lavage samples of the cohorts inoculated with Spn alone against those inoculated with Spn in combination with an antecedent influenza A virus infection and the differences in bacterial and viral titers between complement deficient and WT mice. Fisher exact test analysis was used to examine the differences in frequency of positive blood cultures Spn between complement deficient and WT mice infected with Spn following IAV infection. In all cases, a *P* value of <0.05 was set as the measure of significance.

## Results

### Antecedent IAV infection and complement deficiency impaired Spn and viral clearance

**Figure 1:**
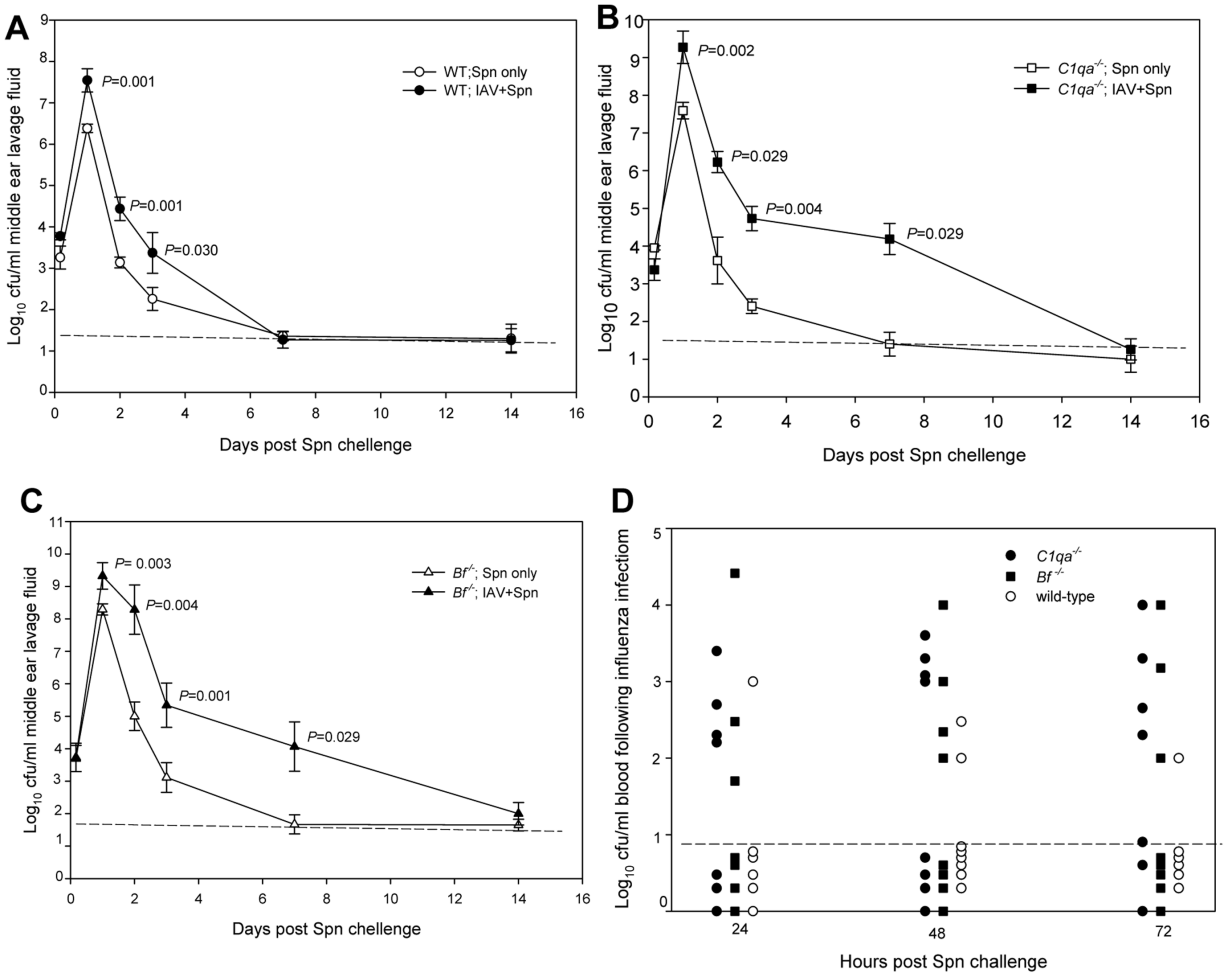
Spn type 6A titers in the middle ear lavage and blood samples. Survival of Spn type 6A in the middle ear of WT (A), *C1qa^−/−^* (B), and *Bf^−/−^* (C) mice infected with Spn with or without a prior influenza virus infection and in the blood sample of mice infected with Spn and IAV(D). Each data point represents the geometric mean of CFU of Spn (± SEM) per milliliter of the middle ear lavage fluid and blood samples. All samples were collected from a total of 6 to 8 animals combined from two separate experiments. Relative *P* values are indicated for each pairwise comparison. The dashed line represents the detection limit of the assay.

Intranasal IAV followed seven days later by transtympanical inoculation of Spn resulted in significantly higher bacterial titers and delayed bacterial clearance from the middle ear compared to Spn alone in WT, *C1qa^−/−^*, and *Bf ^−/−^* mice (Fig. 1 A, B, C). On day 1 post challenge, Spn concentrations in the middle ear lavage samples from cohorts infected with both IAV and Spn were 1.0–1.5 log_10_-fold higher than the groups infected with Spn alone (*P*<0.05). On days 2 and 3 post challenge, the titers remained significantly higher for cohorts infected with both IAV and Spn than Spn alone (*P*<0.05). In the cohorts infected with Spn following IAV infection, the titers of Spn type 6A in the middle ear lavage samples of *C1qa^−/−^* and *Bf^−/−^* were significantly higher than wild-type mice at 24 h (*P* = 0.037 and *P* = 0.007 respectively), 48 h (*P* = 0.024 and *P* = 0.005 respectively), and 72 h (*P* = 0.034 and *P* = 0.002 respectively) post infection. In addition, the clearance kinetics for Spn were markedly different in WT and complement deficient mice. The concentration of Spn 6A remained detectable on day 7 post inoculation in cohorts infected with both IAV and Spn in *C1qa^−/−^* and *Bf ^−/−^* mice. In contrast Spn was eliminated from the middle ear in 7 out 8 WT mice infected with both IAV and Spn (Fig. 1 A, B C).

**Figure 2:**
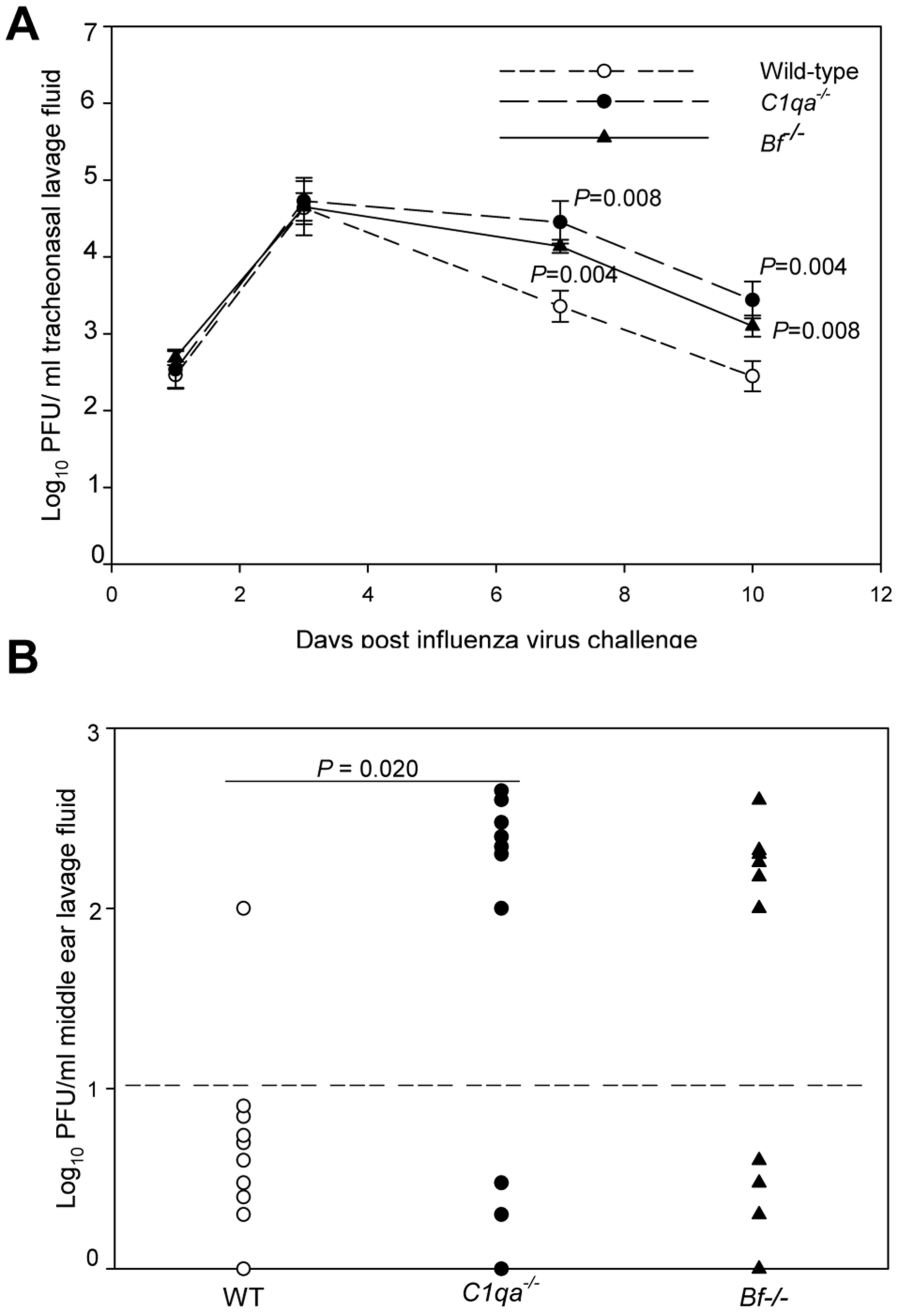
Viral titers in tracheonasal and middle ear lavage samples. Viral clearance from the nasopharynx of WT, *C1qa^−/−^*, and *Bf^−/−^* mice (A) and viral titers on day 7 in middle ear (B). Each data point represents the geometric mean of PFU of IAV (± SEM) per milliliter of the tracheonasal and middle ear lavage fluid. All samples were collected from a total of 10 animals combined from two separate experiments. Relative *P* values are indicated for each comparison with WT mice. The dashed line represents the detection limit of the assay.

Blood culture titers of Spn type 6A for each cohort coinfected with IAV and Spn are shown in Fig. 1D. Persistent bacteremia was noted for all the cohorts of the complement deficient mice across the 24, 48, and 72 h time periods. Spn type 6A was isolated from the blood samples of 54.5% of *C1qa^−/−^*, 43.5% of *Bf ^−/−^*, and 25% of wild-type mice during the 72 h observation period (*P* = 0.047 for *C1qa^−/−^* versus wild-type mice). The frequency of positive blood cultures was not significantly different between *Bf ^−/−^* and *C1qa^−/−^* mice. IAV titers in tracheonasal lavage samples initially increased exponentially reaching maximum titers per ml of lavage samples at 72 h post inoculation (Fig. 2A) for WT, *C1qa^−/−^*, and *Bf^−/−^* mice. However, viral titers remained high through 10 days post infection in *C1qa^−/−^*, and *Bf^−/−^* mice. Viral titers in WT mice declined on days 7 and 10 as the mice began to recover and the titers were significantly lower than C1*^−/−^qa* or *Bf^−/−^* mice. IAV was cleared from the middle ear on day 7 in 9 out 10 WT mice. In contrast, IAV was detected in 7 out 10 *C1^−/−^qa* mice *(P* = 0.020) and 6 out10 *Bf^−/−^* mice (Fig. 2B).

Taken together, these data indicate that deficiencies in C1qa and factor B tended to enhance the susceptibility to IAV and acute pneumococcal OM following influenza.

### Antecedent IAV infection and complement deficiencies facilitated inflammation in the eustachian tube and middle ear

**Figure 3:**
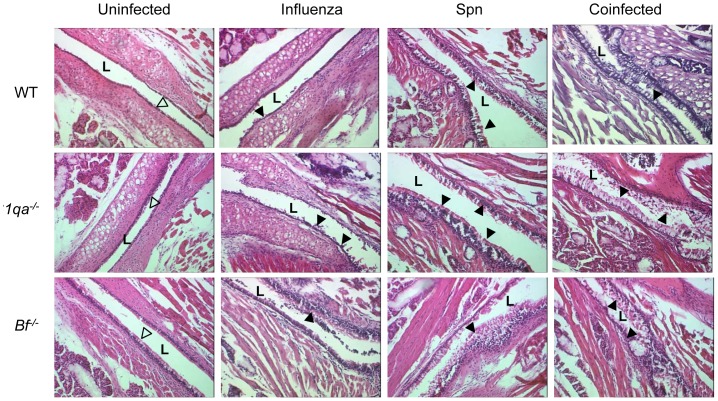
Representative H&E-stained sections of mid-portion of eustachian tube (ET). The structural changes on day 3 after Spn infection in WT, *C1qa^−/−^*, and *Bf ^−/−^* mice infected with influenza virus or either pathogen alone. The tissue damages of ETs in complement deficient mice infected with IAV and Spn were evident (magnification, ×200). Eustachian tube lumen is labeled with “L”. Open arrowheads indicate ciliated mucosa. Filled arrowheads indicate epithelial denudation in IAV infected cohorts, and thickened mucosal epithelium with goblet cell hyperplasia in Spn and coinfected cohorts.

**Figure 4:**
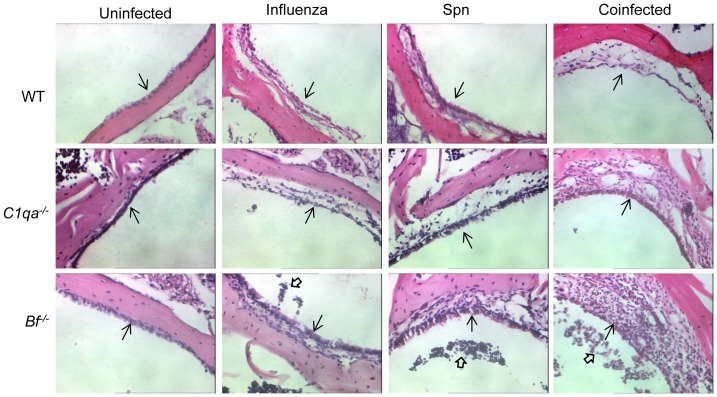
Representative H&E-stained middle ear sections. Middle ear inflammation on day 3 after Spn infection in WT, *C1qa^−/−^*, and *Bf ^−/−^* mice infected with IAV or either pathogen alone. The enhanced inflammatory infiltrates and mucosa thickness in the middle ear epithelium were evident in *C1qa^−/−^* and *Bf ^−/−^* mice infected with IAV and Spn. Open arrowheads indicate inflammatory cell infiltrates, and arrows indicate the middle ear mucosa (magnification, ×200).

Histopathological evaluation revealed that antecedent infection with IAV enhanced eustachian tube and middle ear mucosal inflammation during acute pneumococcal OM. Mock inoculation did not induce structural changes in the ET and middle ear mucosa. On day 3 post Spn inoculation, antecedent IAV infection induced significant structural changes in the ET with fewer ciliated cells and hyperplasia of mucosal epithelium with increased goblet cells and inflammatory cells. C1qa or Bf deficient mice with antecedent IAV infection showed increased ET damage (Fig. 3). More inflammatory epithelium and mucosal thickening were noted in the middle ear space of the antecedent IAV infected cohorts than groups infected with either pathogen alone. The changes in the middle ear mucosa were much more pronounced and persistent in *C1qa^−/−^* and *Bf^−/−^* mice than in wild-type mice (Fig. 4). These data demonstrate increased tissue damage and inflammation in acute pneumococcal OM following influenza virus infection, particularly in mice deficient in the classical or alternative complement pathways.

### Antecedent IAV infection induced enhanced complement activation

**Figure 5:**
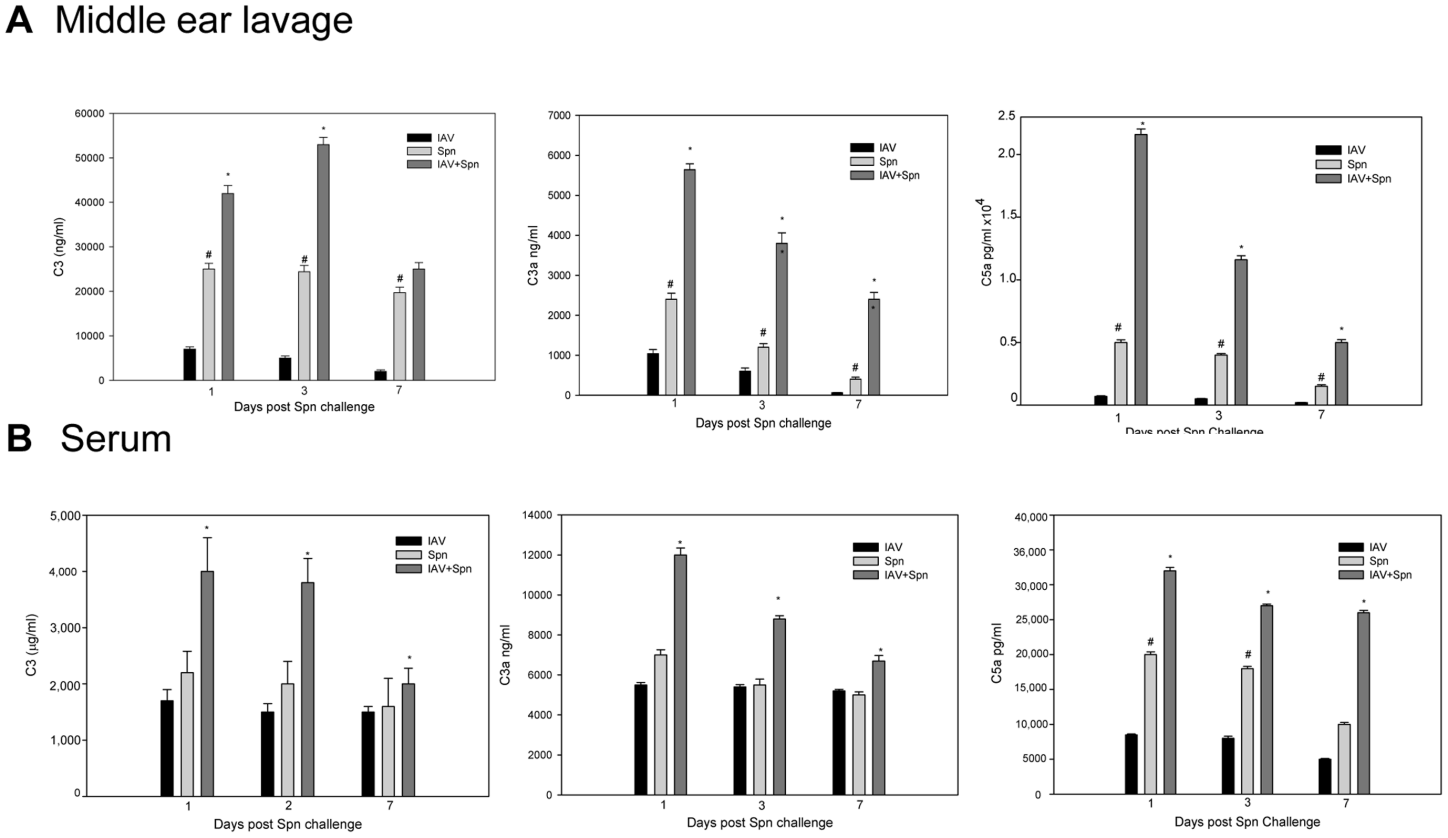
Complement activation during OM. Concentrations of C3, C3a, and C5a in the middle ear lavage fluid samples (A) and serum samples (B) from WT mice infected with a combination of IAV and Spn or either pathogen alone. Results are the mean concentrations (± SEM) in the middle ear lavage and serum samples from 5 mice from two separate experiments. *P<0.05 for the comparison of the combined infection with each single pathogen alone. #, *P*<0.05 for the comparison of mice infected with Spn alone with IAV alone.

**Figure 6:**
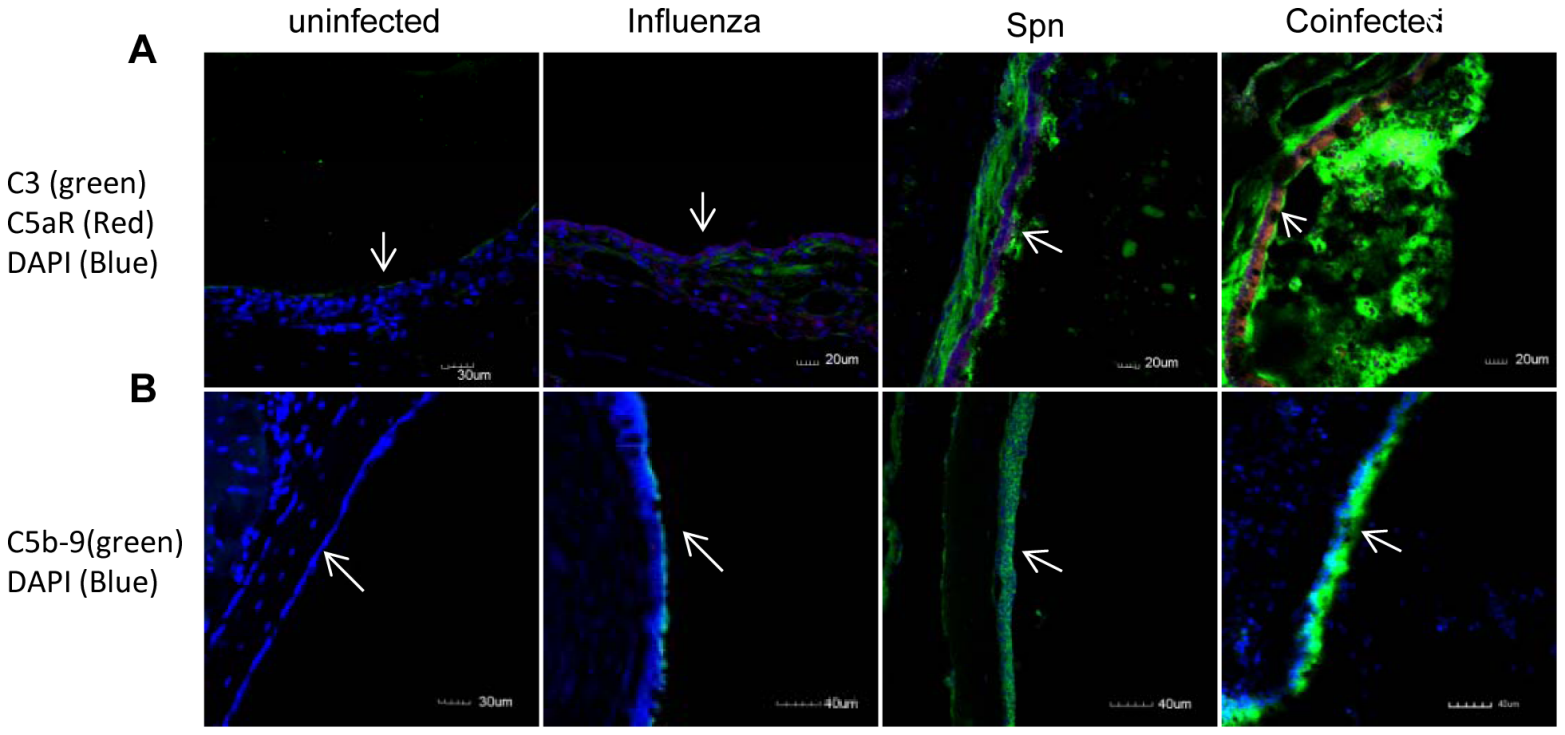
Representative immunofluorescence staining for complement component deposition in the middle ear mucosa. (A) C3 (green) and C5aR (red) deposition in the middle ear mucosa at 24 h post Spn infection. Nucleic acids were stained with DAPI (blue). Faint staining was observed in sham control mice. Increased C3 and C5aR immunofluorescence staining in the middle ear epithelium of mice infected with IAV and Spn was evident. (B). Increased C5b-9 immunofluorescence staining (green) in the middle ear epithelium in mice at 24 after transtympanical inoculation with Spn with a prior IAV infection compared with the sham control and single pathogen infected cohorts. Arrows indicate the middle ear mucosa. Representative imagines from three mice are shown.

We previously demonstrated that the complement alternative pathway and C3 activation in the middle ear account for the local immune response to Spn [18]. Influenza virus is known to induce complement activation [28], [29]. Accordingly we were interested in determining the effect of antecedent IAV infection on the extent of activation of the complement system. We found that the levels of anaphylatoxins C3a and C5a in the middle ear lavage fluid and serum samples of cohorts infected with both IAV and Spn were significantly higher than for mice infected with either pathogen alone (Fig. 5A and 5B). Immunofluorescence staining for the complement component deposition on the middle ear epithelium on day 1 post Spn infection showed increased C3, C5aR (Fig. 6A), and C5b-9 (Fig. 6B) deposition on the inflammatory middle ear epithelium in the cohorts infected with both IAV and Spn compared to those infected with either pathogen alone. These findings indicate that prior IAV infection enhances complement activation during acute pneumococcal OM.

### Deficiency in C5aR reduced severity of acute pneumococcal OM with an antecedent of influenza virus infection

To further assess the functional role of the anaphylatoxin C5a in the pathogenesis of acute pneumococcal OM, we investigated its effect on the severity of OM in mice deficient in C5a receptor (*C5ar1^−/−^*). We first examined whether the complement activation is comparable between

**Figure 7:**
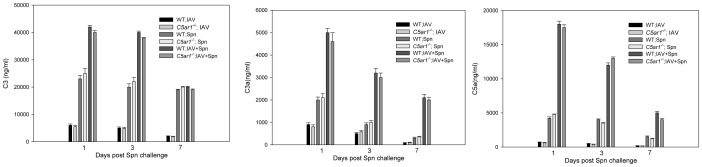
Deletion of C5aR did not affect the complement activation during acute pneumococcal OM. Concentrations of C3, C3a, and C5a in the middle ear lavage samples on days 1, 3, and 7 post Spn infection (corresponding to days 8, 9 and 10 post-viral infection) in WT and *C5ar1^−/−^* mice infected with Spn alone, or Spn with prior IAV, or IAV alone. Results are the mean concentrations (± SEM) in the middle ear lavage pooled from five mice from two separate experiments. There were no significant differences in the concentrations of C3, C3a and C5a between *C5ar1^−/−^* and WT mice.

*C5ar1^−/−^* and its WT control mice during IAV infection or acute pneumococcal OM with or without prior IAV infection. Although the levels of anaphylatoxins C3, C3a, and C5a in the middle ear lavage fluid samples of cohorts infected with both IAV and Spn were significantly higher than for mice infected with either pathogen alone in both *C5ar1^−/−^* and WT mice, there were no significant differences in the levels of C3, C3a and C5a in the middle ear lavage fluid samples of the infected cohorts between *C5ar1^−/−^* and WT mice (Fig. 7). These data indicate that the genetic manipulation in *C5ar1^−/−^* mice does not affect the complement activation.

**Figure 8:**
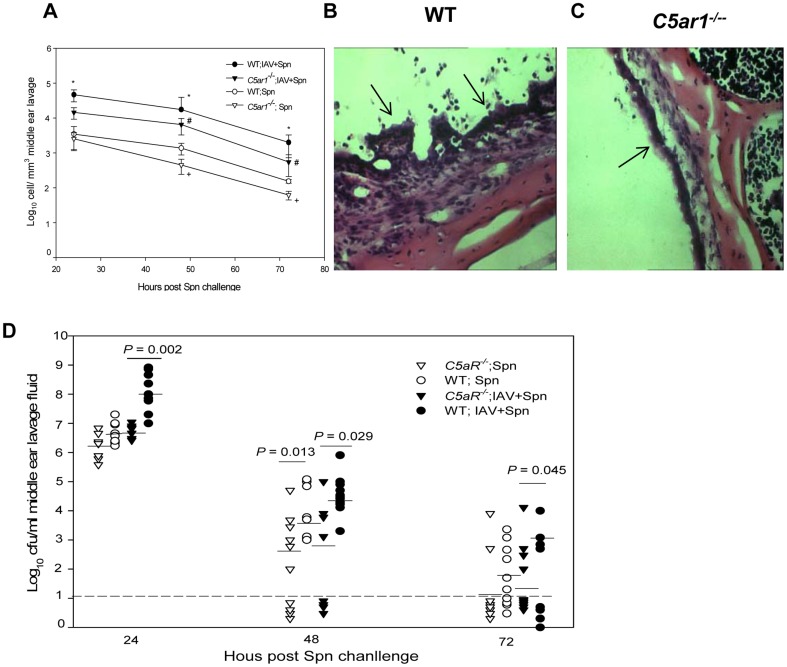
Deficiency in C5aR enhances bacterial clearance and reduces the severity of acute pneumococcal OM. (A). Accumulation of inflammatory cells in the middle ears of mice following transtympanical inoculation of Spn. Each data point represents the mean concentration of inflammatory cells (± SEM) per cubic millimeter of the middle ear lavage fluid. These results were obtained from a total of 6 to 11 animals combined from two separate experiments. *, *P*<0.05 for the comparison of the WT with *C5ar1^−/−^* mice infected with IAV and Spn or WT mice infected with Spn alone. #. *P*<0.05 for the comparison of the *C5ar1^−/−^* mice infection with both pathogens with Spn alone infected C5ar1*^−/−^* mice. +. *P*<0.05 for the comparison of *C5ar1^−/−^* mice infected with Spn alone iwith WT mice. (B) and (C). Representative H&E-stained middle ear sections of the mice at 24 h post Spn infection from coinfected WT (B) and *C5ar1^−/−^* mice (C). Arrows indicate the middle ear mucosa. (D) Survival of Spn type 6A in the middle ear of WT and *C5ar1^−/−^* mice coinfected with influenza virus or mice infected with Spn alone. Each data point represents the mean of CFU of Spn (± SEM) per milliliter of the middle ear lavage fluid samples from a total of 6 to 8 animals combined from two separate experiments. Relative *P* values are indicated for each pairwise comparison. The dashed line represents the detection limit of the assay.

We found significantly more inflammatory cells in the lavage samples collected from WT cohorts infected with both IAV and Spn type 6A than *C5ar1^−/−^* mice at 24, 48, and 72 h post Spn challenge (*P*<0.05) (Fig. 8A). There were more inflammatory cells in the middle ear at 48 and 72 h post Spn infection in mice infected with Spn alone (Fig 8A). Histopathological evaluation showed that at 24 h post inoculation, more inflammatory cells and mucosal thickening were noted in the middle ear space of WT cohorts infected with both IAV and Spn compared to *C5ar1^−/−^* mice (Fig 8B, 8C). Bacterial titers of Spn in the middle ear lavage fluid samples from *C5ar1^−/−^* cohorts infected with both IAV and Spn were significantly lower than in samples from WT mice at 24, 48, and 72 h post Spn infection (*P*<0.05) (Fig 8D). We found no difference in Spn titers between Spn alone infected *C5ar1^−/−^* and WT mice at 24 h post infection. However, Spn were eliminated from the middle ear in 4 out of 10 *C5ar1^−/−^* mice at 48 h post challenge, but in none of the WT mice.

**Figure 9:**
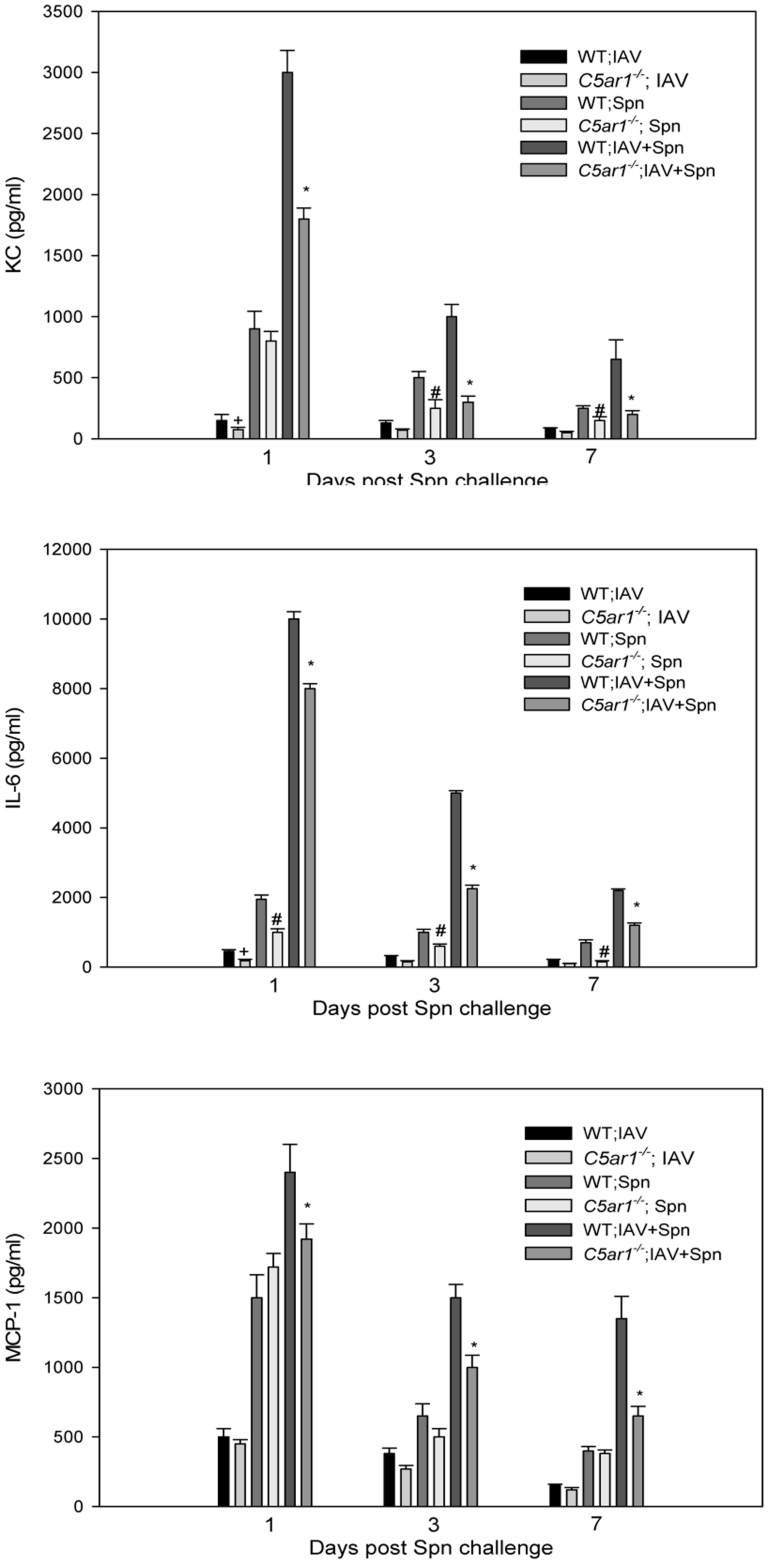
Loss of C5aR reduces the levels of inflammatory mediators in the middle ear. Concentrations of IL-6, mKC, and MCP-1 in the middle ear lavage samples on days 1, 3, and 7 post Spn infection (corresponding to days 8, 9 and 10 post-viral infection) in coinfected WT and *C5ar1^−/−^* mice or mice infected with Spn or IAV alone. Results are the mean concentrations (±S.E.M.) from two separate experiments. *; *P*<0.01 for the comparison of *C5ar1^−/−^* with WT mice infected with IAV and Spn. ^#^; *P*<0.05 for the comparison of Spn alone infected *C5ar1^−/−^* with WT mice. +; *P*<0.05 for the comparison of *C5ar1^−/−^* mice infected with IAV alone with WT mice.

In order to determine whether reduced inflammatory mediator levels in *C5ar1^−/−^* mice infected with IAV and Spn might affect its disease course of secondary acute pneumococcal OM, we measured IL-6, MCP-1, and CXCL1/KC chemokine levels in the middle ear lavage samples obtained at 24, 48, and 72 h post Spn inoculation from mice infected with or without prior IAV infection or with IAV alone. The levels of IL-6 and KC were significantly higher in WT than in *C5ar1^−/−^* mice infected with IAV alone on Day 8 post viral infection (Fig. 9). IL-6, MCP-1, and KC levels were significantly higher in WT mice than in *C5ar1^−/−^* mice infected with both pathogens (*P*<0.01) (Fig. 9). Similarly, IL-6 levels were significantly higher in WT mice than in *C5ar1^−/−^* mice infected with Spn alone (*P*<0.05 respectively).There were no significant differences in the levels of CXCL1/KC among wild-type and *C5ar1^−/−^* mice at 24 h infected with Spn alone, but there were significant differences at 48 and 72 h post infection. These data are consistent with greater influx of inflammatory cells in the middle ears of WT compared to *C5ar1^−/−^* mice. These findings suggest that absence of the C5a-C5aR axis interactions results in an increased bacterial clearance with a decreased inflammatory response and pathology during acute pneumococcal OM following influenza infection.

## Discussion

Reports from our laboratory and others suggest that IAV promotes Spn-induced OM [4], [5], [6], [7]. Clinically, IAV, which infects primarily upper respiratory tract epithelial cells, is closely associated with OM in children, especially those who are carriers of Spn at the time of influenza virus infection [5]. Previous studies of the mechanisms underlying the synergistic interaction between Spn and influenza A virus have focused primarily on the role of viral compromising the eustachian tube, mucosal integrity, and tubal function in the chinchilla OM model. Although the chinchilla OM model has been used for the study of antecedent virus-bacterial infections because it resembles the natural route of infection in humans, the lack of chinchilla strains with targeted gene disruption and specific immunological reagents limits our ability to use the model to dissect the contribution of important components of the host's immune response to the pathogens. The mouse is a useful animal model for induction of middle ear infection. The method of choice for OM induction, intranasal (i.n.) or direct middle ear administration of pathogens, depends on the experimental needs of the investigation. Induction of OM using an i.n. challenge model is preferred for studies that involve evaluating protective immunity against nasopharyngeal colonization and subsequent infection in the middle ear. Direct middle ear challenge provides the most direct approach to evaluating the role of protective immunity against middle ear infection or resolution of OM [30]. In the current dual infection model, we used sublethal doses of both pathogens since the majority of pediatric patients with OM do not have fatal infections. We demonstrated that following IAV infection, Spn produced significant structural changes in the ET and middle ear mucosa, increased complement activation, higher titers of Spn, and inflammatory mediators in the middle ear compared to Spn alone. Thus our model can be used to elucidate the synergistic molecular mechanisms during post-influenza acute pneumococcal OM.

We previously reported that C3 activation and opsonophagocytosis of Spn were greatly attenuated in C1qa and factor B deficient mice, which contributes to the enhanced susceptibility of these mice to acute pneumococcal OM [17], [18]. In the current study we were able to show that C1qa and factor B of the complement components play an essential role in viral and Spn clearance during acute pneumococcal OM following IAV infection. One of the interesting findings in this study was that following IAV infection C1qa and Bf deficient mice not only had high levels of Spn in the middle ear, but also developed ET structural damage with a delayed IAV clearance from the nasopharynx and middle ear that could further facilitate the severity of OM. It has been reported that the complement classical pathway components C1, C3 and C4 are required for neutralization of influenza virus [31], [32], but as of yet the role of the alternative pathway in this process is not clear. In the current study, we demonstrated that factor B also plays a protective role at least equal to C1qa in the middle ear defense against Spn during IAV infection. Factor B is one of the components of the alternative pathway and can be activated by microbial fragments. Spontaneously hydrolyzed C3 complexes with factor B and then is cleaved by factor D to generate C3bBb, the alternative pathway C3 convertase that cleaves C3 to C3b. In addition, factor B amplifies complement activation initiated through the classical/lectin pathways. Multiple mechanisms appear to be responsible for the enhanced susceptibility of C1qa and Bf deficient mice, following IAV infection. Further studies are needed to understand the mechanisms whereby influenza A virus enhances susceptibility and the pathogenesis of pneumococcal OM.

OM is characterized by inflammation of the middle ear with the predominance of polymorphonuclear neutrophil (PMN) in the early stage, and lymphocytes and other mononuclear cells in the late stage of OM along with the release of numerous proinflammatory cytokines and chemokines [33], [34], [35], [36]. Inflammation resolution during OM requires not only eradication of bacteria or viruses by innate and adaptive immune mechanisms but also a tight regulation of the inflammatory responses in the middle ear space. Loss of control and consequent exaggeration and prolongation of the inflammatory response can lead to tissue injury in the middle ear cleft. In the present study, we showed that antecedent IAV infection induced enhanced complement activation in the middle ear and serum in Spn infected mice. This was demonstrated by the increased levels of C3, C3a, and C5a. Immunostaining for C3, C5aR, and C5b-9 showed intense deposition in the middle ear epithelium in mice during IAV infection. Complement components including C3a, C5a, and sC5b-9 have been detected in middle ear fluid and mucosa in both clinical and experimental infections [37], [38]. Intense complement activation has been demonstrated in chronic otitis media with effusion (OME) in association with persistent inflammation in the middle ear [39]. We recently reported that levels of C3a, C5a, and sC5b-9 in the middle ear are informative biomarkers for persistent inflammation during recurrent acute OM in children [40]. It is well known that C5a binding to C5aR or C5L2 exerts a range of biological effects including the chemoattraction of inflammatory cells and induction of the “cytokine storm” during the inflammatory response [41]. The effects elicited by C5a/C5aR interaction appear to be organ and cell-type-specific [42].

In the current study, we use *C5ar1^−/−^* mice to determine the functional role of these interactions in the development of secondary acute pneumococcal OM following influenza infection. We observed that levels of IL-6, mKC, and MCP-1 were substantially reduced, along with reduced inflammatory cell recruitment, mucosal inflammation and improved bacterial clearance in the middle ear of *C5ar1^−/−^* mice. We think that the deletion of C5aR contributes to the reduced proinflammatory immune responses resulting in increased Spn clearance and augmented resolution of inflammation in response to Spn and IAV in our model. In this regard, Short et al. demonstrated that the enhanced inflammatory response induced by IAV facilitates pneumococcal growth and subsequent episodes of acute OM [14]. Others suggested that an immunopathologic response such as inflammation-mediated epithelial denudation or a viral preoccupation of the innate immune system may provide a more hospitable environment for pneumococcal outgrowth and disease in influenza virus-pneumococcus synergy [43]. Further investigations are required to understand the specific inflammatory components responsible for increased bacterial replication and disease [43].

In the current study we focused on C5aR without specifically investigating the role of C5L2. C5L2 has been shown to play a proinflammatory role during sepsis through intracellular signaling, release of cytokines IL-6, tumor necrosis factor α (TNF-α), and high mobility group box 1(HMGB1) [44]. C5L2 also plays an anti-inflammatory role as a recycling decoy receptor or via β-arrestin pathway [44]. In this study, although the level of IL-6 in the middle ear was statistically lower in *C5ar1^−/−^* than WT mice, the high level of IL-6 on day 1 post Spn infection in the coinfection cohort might be attributed to the modulation of C5L2. The expression and function of C5L2 warrant further investigation, especially the possible attenuation or propagation of inflammation during acute pneumococcal OM.

To the best of our knowledge, the current report is the first to present the role of C5a/C5aR interactions in acute pneumococcal OM pathogenesis. Our data are consistent with a previous study showing that a C5 deficiency and C5a blockade protects mice against pneumococcal meningitis [45]. Therapeutic interventions using neutralizing antibodies against C5a or C5aR and C5aR antagonists remain attractive targets for human disease models such as sepsis, cancer, inflammatory bowel disease, arthritis, and pregnancy-related complications [46], [47]. Our findings suggest that the targeting of the C5a and C5aR axis may have therapeutic potential for OM and that this targeting warrants further investigation.

In conclusion, our data indicate that deficiencies in the complement classical and alternative pathways contribute to enhanced susceptibility to acute pneumococcal OM following infection with IAV. The complement component C5a in the middle ear induced following IAV infection and Spn facilitates the severity of acute pneumococcal OM. Using C5aR deficient mice we demonstrated that the middle ear inflammatory responses were attenuated during acute pneumococcal OM with prior IAV infection. These findings suggest that pharmacological targeting of the C5a-C5aR axis might attenuate the progression of acute pneumococcal OM following influenza virus infection.
